# An EPR study of the cognitive processes underlying the impact of self-relevant information on emotional word processing

**DOI:** 10.1186/s40359-024-01586-z

**Published:** 2024-02-22

**Authors:** Ping Zhang, Yidan Song, Endale Tadesse, Sabika Khalid, Chunhai Gao, Weijun Li

**Affiliations:** 1 Shanghai Urban Construction Vocational College, Shanghai, China; 2 Research Center of Brain and Cognitive Neuroscience, Liaoning Normal University, Dalian, China; 3 Key Laboratory of Brain and Cognitive Neuroscience, Dalian, Liaoning Province China; 4 Faculty of Education, Zhejiang Normal University, Jinhua, China; 5 Faculty of Education, Shenzhen University, 3688 Nanhai Avenue, Nanshan District, Shenzhen, China

**Keywords:** Self-negative bias, Self-positive bias, Emotional words, Subthreshold, Suprathreshold

## Abstract

Using the event-related potentials (ERPs) technique, this study successively presented names (in either a supra- or subthreshold manner) and emotional words to examine how self-relevant cue (self-name) affects emotional word processing in word class judgment task (to determine whether an emotional word is a noun or adjective) and valence judgment task (to determine whether an emotional word is positive or negative). At the suprathreshold condition, self-relevant positive words elicited a more significant Early posterior negativity (EPN) than negative words only in the valence judgment task. In contrast, at the subthreshold condition, self-relevant negative words elicited an enhanced Late positive potential (LPP) than positive words only in the word class judgment task. These results indicate that self-relevant cue affects emotional word processing at both suprathreshold and subthreshold conditions; nevertheless, the effect manifests as self-positive bias at the suprathreshold condition and self-negative bias at the subthreshold condition. The experimental task modulates these dynamics.

## Introduction

In social interactions, stimuli with high sociality or adaptive significance can instantly capture attention. Early in life, humans prioritized self-relevant information [1–3]. People’s preference for self-relevant information develops their social cognition. Likewise, humans’ rapid processing of emotional information helps them adapt their behavior to the environment, which is essential for human survival, development, and social adaptation. Importantly, emotional information often accompanies person-related information, and self-relevant emotional stimuli can stimulate individuals’ attention. Therefore, exploring the complex dynamic process of self-affecting emotional information processing is necessary and valuable. The present study intended to explore this question using ERPs in both supra- and subthreshold conditions.

A person’s name, an abstract part of the self, has been proven to be closely related to the “inner self.” Self-name is unique in terms of emotional value and psychological meaning and has advantages in cognitive processing. For example, self-name can attract immediate attention and be rapidly processed [1], and the mere hearing of one’s name can fully activate their self-concept [2, 3]. Recently, researchers have confirmed via functional magnetic resonance imaging (fMRI) that there were different neural bases for processing self-name and non-self-name [4, 5]. Moreover, based on event-related potentials (ERPs), researchers have found that processing a self-name has priority in both the early (P2 and N250) and late (P300) stages of experimental tasks [6–8]. Subthreshold priming is presenting a priming stimulus for a short duration with low intensity to facilitate subsequent processing of the target stimulus despite a participant’s lack of awareness [9]. It has been found that both supra- and subthreshold presentations of self-name activate the individual’s self-concept, leading to more automatic responses to the target stimulus [10].

Furthermore, there is also research explicitly investigating the mechanisms of self-reference effect. After measuring brain activity during the self-referential effect using event-related fMRI technology, Yaoi and Osaka proposed that the principle behind the self-referential effect is the enhancement of memory traces that influence brain processing [11]. Nonetheless, Wang et al.‘s study (2019) found that the self-referential effect only improves mnemonic efficiency and does not enhance the accuracy of memory [12].

Emotional information can be transmitted in different ways, of which emotional faces are the most direct carriers. Although ERPs have been used for a long time to explore the internal neural processes of emotional perception, recognition, and response in healthy individuals [13–15], researchers have begun to focus on impaired emotional perception and emotion regulation in people with emotional disorders. For example, researchers have examined the response of patients with emotional disorders to emotional faces and found that there are defects in the unconscious processing of patients with major depression; they are susceptible to negative emotional cues or events, and their response to positive emotional stimuli is relatively weakened [16]. It was also found that women with borderline personality disorder had a poor ability to distinguish facial pleasure [17]. The high sensitivity of individuals with high social anxiety to threatening expressions is related to their strong structural coding and fine processing [18]. Furthermore, researchers have found that individuals with severe depression exhibit a self-negative attribution bias in emotional word tasks [19].

Likewise, emotional pictures and words can carry and convey emotional information and elicit emotional responses [20]. However, emotional words are abstract symbols that are visual but lack obvious emotional cues, and their emotional connotations must be acquired through semantic processing. Researchers have found that emotional words are perceived faster and attract more attention than neutral words [21]. Relevant ERP studies show that emotional words can trigger emotional effects in early and late processing. In the early stage, the emotional features of the stimulus are quickly identified. Compared with positive and neutral words, negative words usually induce P1 with a smaller amplitude [22, 23] and N1 with a larger amplitude [24]. Some studies have also found that positive words induce P2 with larger amplitude than negative and neutral words [25, 26]. Besides, emotional words elicit more significant Early posterior negativity (EPN) amplitude than neutral words. This indicates that emotional words gain selective attention after early perceptual encoding [27].

In the later processing stage, emotional word processing produces complex results, reflected in the N400 and the Late positive potential (LPP) amplitude. The N400 is sensitive to the semantic processing of stimuli, and larger N400 amplitudes reflect the violation of semantic expectations and difficulties in resource integration [28]. Furthermore, the N400 amplitude increases in response to the emotional salience of words, reflecting a unique aspect of emotional semantic processing, and the results are mixed [24, 29, 30]. The LPP, which usually appears within 500–800 ms after stimulus presentation, is an effective indicator of continuous stimulus processing and encoding, modulated by the emotional content of the stimulus [31]. A study found that emotional words elicited larger LPP amplitudes than neutral words [32], while another study found that negative words, compared with positive words, elicited larger LPP amplitudes [33]. However, a contrary result was observed in other research, where positive words elicited larger LPP amplitudes [34].

From the aforementioned studies, it is evident that the processing of emotional words yields complex and diverse results. Aside from the inherent characteristics of the stimuli, these results are also influenced by other factors, such as self-relevant cues. In reality, emotional information is often integrated with self-relevant information, and there is a complex interaction of self, general cognition, and emotion [35, 36]. Relevant ERP studies have found that self-relevant cues (i.e., self-name) affect emotional word processing, but different paradigms and materials have produced inconsistent results. When using explicit self-referential evaluation tasks, such as judging how well trait words describe the self [19, 37] and completing tasks on emotional sentence comprehension [38], results of self-positive bias were often obtained (reflected in ERP results as self-relevant positive words eliciting smaller N400 amplitudes or larger LPP amplitudes than that elicited by negative comments, as well as self-relevant positive words producing larger LPP amplitudes than that elicited by non-self-relevant positive words). This may stem from individuals exhibiting a self-positive attribution bias, attributing positive traits or outcomes to stable internal personal characteristics while considering negative traits or outcomes unrelated to their traits [19, 39]. Another consideration is that individuals associate the self with positively valenced stimuli rather than negatively valenced stimuli, which may be driven by implicit associations between the self and positivity, that is, better performance in the implicit association task when the self and positivity are grouped [40–42]. However, the results are not necessarily self-positively biased when participants are asked to attend to or process non-emotional information through implicit tasks such as judging kanji order [43, 44]. They may even be self-negatively biased [45, 46]. In short, self-relevant cues affect emotional word processing, but whether this is reflected in a positive or negative bias remains unclear.

Regarding the interaction between self and emotional information, different researchers have proposed theories to explain the possible relationship between them, such as the emotion appraisal theory [47, 48], as well as the Activation Association Theory (AAT) and Activation/Monitoring Theory (AMT]) [49, 50]. As relevant to the present study, the emotion appraisal theory explains the interaction between emotional information and the self. According to this theory, individuals first evaluate the relevance of the stimuli to themselves, then assess the impact of the stimuli on their well-being, and finally evaluate their ability to cope with these stimuli [47, 48].

Recent research has shown that self and emotions are regulated mainly by unconscious, implicit processes [51]. Subthreshold presentation of self-relevant cues instantly stimulates processes that compare the self with others, leading to self-evaluation [52]. It needs to be clarified how self-information, presented in supra- and subthreshold ways, affects emotional information processing. Furthermore, previous research has shown that the depth of emotional word processing affects individuals’ recognition of their or others’ emotions [53, 54]. The inconsistent results of positive/negative self-bias mentioned above may stem from the different degrees of semantic acquisition of expressive words, i.e., the experiments used various tasks [37–46].

Thus, we employed an ERP technique using self-names and non-self-names as self-relevance cues, presenting names (suprathreshold or subthreshold) and emotional words (positive or negative) successively to examine the dynamic time course of self-relevance affecting emotional word processing in both lexical judgments (to determine whether the expressive words are nouns or adjectives) and valence judgment (to determine whether the emotional words are positive or negative) tasks. We expected subjects to successfully distinguish between themselves and others at the sub- and suprathreshold conditions and regulate the processing of emotional information. Regardless of the study, subthreshold priming causes individuals to perceive self-cues unconsciously, which leads to more automatic responses to the target stimulus, manifested as self-negative bias [45, 55]. When presenting suprathreshold self-information, subjects may show self-positive preference in an explicit emotion task (valence judgments) [35, 36] and a self-negative bias in lexical decisions of implicitly processed emotional information [45, 46], according to previous related studies.

## Method

### Participants

The sample size was based on power analyses carried out with G-power [56]. In order to detect medium effect sizes (Cohen’s *f* = 0.25) with 80% power for threshold level × task types × self-relevant cue × emotion valence ANOVAs, a minimum of 11 participants were required. Based on this, we recruited much more participants. Twenty-four college students (10 males, aged between 19 and 26, *M*_age_ = 21.8, *SD* = 2.4) were paid to participate in this experiment. All the participants were right-handed and were native Chinese speakers who had normal or corrected to normal vision. They were physically healthy, with no mental illness, emotional disorder, or family history of genetic predisposition. Participants had never participated in similar experiments. The participants completed the state-trait anxiety questionnaire (STAI) and the Beck depression inventory (BDI) before the investigation; all scores were within the normal range. The participants signed informed consent forms before they became part of the experiment. In addition, the study was approved by the ethics committee of Liaoning Normal University and is in accordance with the Declaration of Helsinki.

The experimental materials consisted of name stimuli, masking stimuli, and emotional words. First, the participant’s name was used as the priming stimulus in the self condition, and the name of a same-sex friend (obtained and programmed before the experiment)—with whom the subject had the best relationship in the last two months—was used as the priming stimulus in the non-self condition. All name stimuli included three-character characters. The masking stimulus consisted of three random Chinese characters (e.g., 迫币输, press/coin/lose 160). Another 320 two-character emotional words from the Chinese emotional word system [57] were selected as target stimuli, including 160 positive and 160 negative words, and each valence included 80 nouns and 80 adjectives. We screened the masking stimuli through a series of steps to guarantee their meanings and valences were controlled to prevent interference with the processing of the targets. They were first randomly formed into Chinese character combinations by a computer, and then two experimenters with a native Chinese language background rated the materials by subjective evaluation, and those containing validity, meaning, or controversial materials were deleted. Finally, these materials were further approved by two other experimenters with a native Chinese language background.

**Table 1:** Mean and standard deviation of valence, arousal, specificity, and word frequency scores

		Positive (M ± SD)	Negative (M ± SD)
Valence	Group 1	6.81 ± 0.31	3.18 ± 0.46
	Group 2	6.80 ± 0.29	3.20 ± 0.40
Arousal	Group 1	5.10 ± 0.74	5.19 ± 0.73
	Group 2	5.03 ± 0.66	5.10 ± 0.78
Specificity	Group 1	3.37 ± 1.10	3.41 ± 0.99
	Group 2	3.48 ± 1.08	3.42 ± 0.99
Word Frequency	Group 1	22.61 ± 27.03	22.55 ± 30.40
	Group 2	23.10 ± 22.38	22.80 ± 41.53

Before the experiment, all emotional words were divided into two homogeneous groups (160 words in each group, including 40 positive nouns, 40 positive adjectives, 40 negative nouns, and 40 negative adjectives). A two-factor repeated measures ANOVA was performed using IBM SPSS 22 with valence (positive and negative) and group (groups 1 and 2) as independent factors and valence, arousal, specificity, and word frequency of the two groups of materials as dependent factors. Among the valence scores, 1 indicated extremely unhappy, annoyed, dissatisfied, sad, and disappointed after reading the word, and nine indicated extremely happy, pleasant, satisfied, and hopeful; arousal, specificity, and word frequency were evaluated with a similar procedure as that of valence, using a 9-point scale. The results revealed that the emotional words differed significantly only on valence scores (positive: 6.80 ± 0.29; negative: 3.19 ± 0.43, *F* (1, 79) = 7702.86, *p* < 0.001, *η*_*p*_^*2*^ = 0.99), but not on other scores (arousal, specificity, and word frequency; *p*s > 0.1). The main effect of the group and the interaction effect of the valence × group were insignificant on all the scores (*ps* > 0.1). These materials were used in the two tasks and presented in both the supra- and subthreshold conditions. The specific scores of the two groups of materials are shown in Table 1.

### Procedure

The experimental procedure consisted of a pre-test and a formal experiment, both conducted using E-Prime 2.0 (Parameters are as follows: the “Refresh Rate Requested” is 60; the “Minimum Acceptable Refresh Rate” is 59; the “Maximum Acceptable Refresh Rate” is 61). In the pre-test, 16 participants (7 males, *M* = 23.7, *SD* = 1.6) who did not participate in the EEG experiment were recruited to determine the threshold level of the self-name. In the pre-test, we varied the presentation time of the self-name from 10 ms to 45 ms in increasing order of equal variance every 3 ms, with ten continuous trials presented at each time setting. The pre-test determined the subthreshold condition for the self-name to be 33 ms with 50% visibility, followed by a 200 ms post-masking stimulus. This time was determined primarily by the following criterion: when the subject answered accurately or vaguely, “It seems to be my name.“.

The formal experiment was conducted in a quiet, comfortable room with soft light. The participants were seated 60 cm before a Liquid Crystal Display (LCD, 23 inches, with a refresh rate of 60 Hz). Each participant completed the valence and word class judgment tasks in sub- and suprathreshold conditions. The subthreshold requirement was always met first, followed by the suprathreshold state. The order of valence and word class judgment tasks was counterbalanced. Before starting the subthreshold condition, participants were informed that they would see rapidly presented Chinese characters and an emotional word and would need to judge the lexicality or valence of the emotive word by pressing a key; they were not informed that the stimulus masked by the Chinese characters was a name. Before starting the suprathreshold task, participants were told that they would see their own or another person’s name, a three-character sequence, and an emotional word, and they would need to judge the word class or valence of emotional words by pressing a key.

**Fig. 1:**
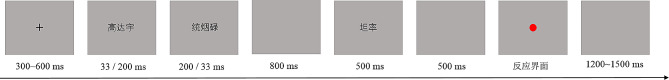
Schematic illustration of a single trial in the experiment. The first number in the time duration of name and masking stimuli presentation represents the subthreshold condition, and the second represents the suprathreshold condition

The formal experiment consisted of two sessions. In the first session, the name stimuli were presented in a subthreshold manner (33 ms). It included valence judgment and word class judgment tasks, which were given with the order counterbalanced among participants. There were 160 trials in each session, including 40 self-positive trials, 40 self-negative trials, 40 other-positive trials, and 40 other-negative trials. All trials were presented in a completely randomized order. Apart from the task differences, the specific process of the presentation was the same for all tests (see Fig. 1): first, the fixation “+” was presented between 300 and 600 ms at the center of the screen, followed by the participant’s name or a friend’s name for a 33 ms duration, then the masking stimulus was presented for 200 ms, followed by a blank screen for 800 ms. Subsequently, an emotional word was presented for 500 ms, followed by another blank screen for 500 ms. Then, the response interface was presented and disappeared after pressing the button; otherwise, it automatically disappeared without a response within 2000 ms. They were finally followed by a blank screen for 1200 to 1500 ms a blank screen. During the response interface, the participants were required to judge the word class or valence of the words according to the task requirements by pressing a key (*F* for positive/noun; *J* for negative/adjective). The key press was balanced between participants. Participants were allowed to rest between the two tasks for as long as they wanted. There were ten practice trials (which could be repeated) before the formal experiments, and participants familiar with the tasks were allowed to perform the formal experiments. After the first part of the subthreshold condition, participants were asked to report the three-character stimulus seen during the investigation. If the participants could not write their names or a friend’s name, the purpose of presenting characters in a subthreshold manner was achieved. Otherwise, the participant’s data were invalid, and they could not participate in subsequent experiments.

In the second session, the name stimuli were presented at 200 ms in a suprathreshold manner. The experimental procedure was the same as the subthreshold session, except that the name stimulus was presented for 200 ms, and the post-masking stimulus was presented for 33 ms; participants were also required to complete two tasks, and task order and key pressing were counterbalanced among participants.

### Data collection and analysis

EEG signals were recorded using an ANT device (ANT Neuro EEGO Inc., Germany) with 64 channels according to the International 10–20 system extension. The signal was recorded at a sampling rate of 500 Hz, with CPz as the online reference. Electrodes M1 and M2 were placed on the left and right mastoids. The vertical electrooculogram (EOG) was recorded from electrodes above and below the left eye. The horizontal EOG was recorded using electrodes placed approximately 1 cm laterally to the outer canthi of both eyes. The impedance between the electrodes and the scalp was lower than 5 kΩ, and the filtered bandpass for online recording was 0.01 to 100 Hz. For offline analysis, the average of the bilateral mastoids was subtracted from the EEG data of each lead for re-referencing. The EEG was corrected for ocular artifacts using the Independent Component Analysis (ICA), and both the EEG epoch for the artifacts and incorrect responses were excluded from the analysis. The EEG signals were bandpass filtered with a high pass of 0.01 Hz and a low key of 30 Hz. The EEG analysis was locked at the onset of the emotional word presentation, and the data were segmented 200 ms before and 800 ms after the beginning of the emotional word. Baseline correction was performed for the segmented data, after which the artifacts (amplitude exceeding ± 80 µv) were removed from the segmented data. Finally, the grand average was performed for the adequate trials retained under each condition.

The corresponding electrodes and time windows were selected according to the observation of grand average waveforms of the EEG data combined with the previous study. The electrodes selected for N1 (80-130ms), P2 (150-250ms) and N400 (300-400ms) included F3, Fz, F4, FC3, FCz, FC4 [58–60]. The electrodes selected for EPN (150-220ms) included P7, PO7, O1, O2, PO8, and P8 [27, 46], and the electrodes selected for LPP (450 ~ 650ms) included C3, Cz, C4, CP3, CPz, CP4 [8, 43]. The average wave amplitudes on these EEG components were analyzed by repeated measures ANOVA using IBM SPSS 22.0. Factors that were subjected to repeated measures ANOVA for behavioral and EEG data in suprathreshold and subthreshold conditions included self-relevance (self vs. non-self), emotional valence (positive vs. negative), and task type (word-class judgment vs. valence judgment). The Greenhouse-Geisser method was used to correct the degrees of freedom in the ANOVA whenever sphericity assumptions were violated [61]. The *p*-values were corrected using Bonferroni in all post hoc comparisons. We adopted a significance level of 0.05 and reported *η*_*p*_^*2*^ as the effect size estimate.

## Results

### Behavioral results

**Fig. 2:**
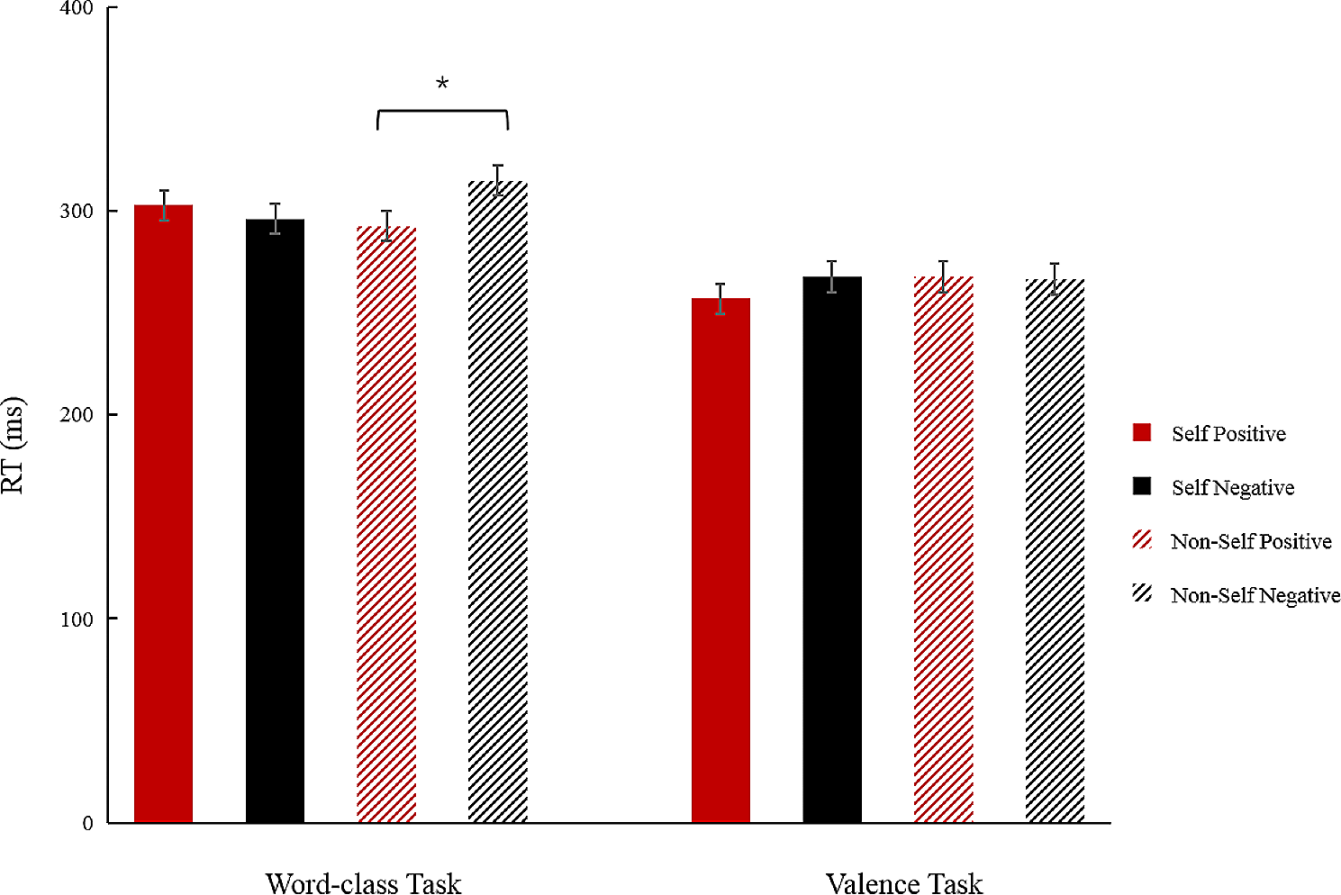
Interaction between Task type × Self-relevant cue × Emotion valence in response time (*<0.05)

A four-factor repeated measures ANOVA of 2 (threshold level: suprathreshold vs. subthreshold) × 2 (task types: lexical task vs. valence task) × 2 (self-relevant cue: self-name vs. non-self name) × 2 (emotion valence: positive vs. negative) revealed a significant main effect of threshold level on Reaction Time (RT) (*F*(1, 23) = 11.10, *p* = 0.003, *η*_*p*_^*2*^ = 0.33). The RT for the suprathreshold condition was faster than the subthreshold condition. The main effect of task type was significant (*F*(1, 23) = 1.69, *p* = 0.011, *η*_*p*_^*2*^ = 0.25). The RT for the valence task was faster than the word-class task (see Fig. 2). The interaction of task type × self-relevant cue × emotion valence was significant (*F*(1, 23) = 7.45, *p* = 0.012, *η*_*p*_^*2*^ = 0.25). Further simple effect analyses revealed a trend of differences between positive and negative words in the self-name condition in valence task, that is, the RT for judging self-relevant positive words were faster (*F*(1, 23) = 4.10, *p* = 0.055, *η*_*p*_^*2*^ = 0.15); the difference between positive and negative words in non-self name condition was not significant (*p* = 0.890); in the word-class task, the RT for positive words, compared with negative words, in the non-self name condition was faster(*F*(1, 23) = 7.27, *p* = 0.013, *η*_*p*_^*2*^ = 0.24). The difference between positive and negative words in the self-name condition was not significant (*p* = 0.346)(see Table 2).

**Table 2:** Mean and standard deviation of response time and accuracy (*M* ± *SD*)

Threshold	Task Type		M ± SD							
			RT (ms)				ACC (%)			
			Positive		Negative		Positive		Negative	
suprathreshold	Valence judgment	Self	245.9 ± 80.5		249.1 ± 101.8		96.2 ± 4.1		94.0 ± 4.0	
		Non-self	248.4 ± 97.2		272.4 ± 95.1		97.1 ± 3.6		93.5 ± 5.5	
	World-class judgment	Self	266.5 ± 73.9		252.8 ± 76.2		90.0 ± 5.9		92.5 ± 5.9	
		Non-self	259.1 ± 79.1		277.0 ± 96.6		92.7 ± 5.9		87.3 ± 6.7	
subthreshold	Valence judgment	Self	267.9 ± 98.8		286.4 ± 110.1		97.6 ± 2.5		93.5 ± 3.4	
		Non-self	286.9 ± 118.2		290.8 ± 113.5		97.2 ± 2.5		92.4 ± 3.9	
	World-class judgment	Self	338.9 ± 107.6		339.5 ± 104.0		89.6 ± 6.3		90.4 ± 6.9	
		Non-self	325.9 ± 66.3		352.7 ± 105.4		91.2 ± 6.8		85.0 ± 6.5	

A four-factor repeated measures ANOVA on Accuracy (ACC) revealed a significant main effect of task type (*F* (1, 23) = 30.69, *p* < 0.001, *η*_*p*_^*2*^ = 0.58). The accuracy of the valence task was higher than the word-class task. The main effect of self-relevant cues was significant (*F* (1, 23) = 9.53, *p* = 0.005, *η*_*p*_^*2*^ = 0.29). The accuracy of the self-name condition was higher than that of the non-self-name condition. The main effect of emotion valence was significant (*F* (1, 23) = 35.91, *p* < 0.001, *η*_*p*_^*2*^ = 0.61). The accuracy of judging positive words was significantly higher than negative ones. The interaction of task type × emotion valence was significant (*F* (1, 23) = 4.79, *p* = 0.039, *η*_*p*_^*2*^ = 0.17). Further simple effect analyses revealed that the accuracy of judging positive words was significantly higher than that of judging negative words in both tasks (valence task: *F* (1, 23) = 26.54, *p* < 0.001, *η*_*p*_^*2*^ = 0.54; word-class task: *F* (1, 23) = 20.07, *p* < 0.001, *η*_*p*_^*2*^ = 0.47). Additionally, the accuracy of judging emotional words was higher in the valence task compared with the word-class task (positive: *F* (1, 23) = 45.60, *p* < 0.001, *η*_*p*_^*2*^ = 0.67; negative: *F* (1, 23) = 15.94, *p* = 0.001, *η*_*p*_^*2*^ = 0.41). The interaction of self-relevant cue × emotion valence was significant (*F* (1, 23) = 36.42, *p* < 0.001, *η*_*p*_^*2*^ = 0.61). Simple effect analyses revealed that the accuracy of judging self-relevant positive words was lower than that of judging non-self-relevant positive words (*F* (1, 23) = 10.57, *p* = 0.004, *η*_*p*_^*2*^ = 0.32). In contrast, the accuracy of judging self-relevant negative words was higher than that of judging non-self-relevant negative words (*F* (1, 23) = 30.40, *p* < 0.001, *η*_*p*_^*2*^ = 0.57).

**Fig. 3:**
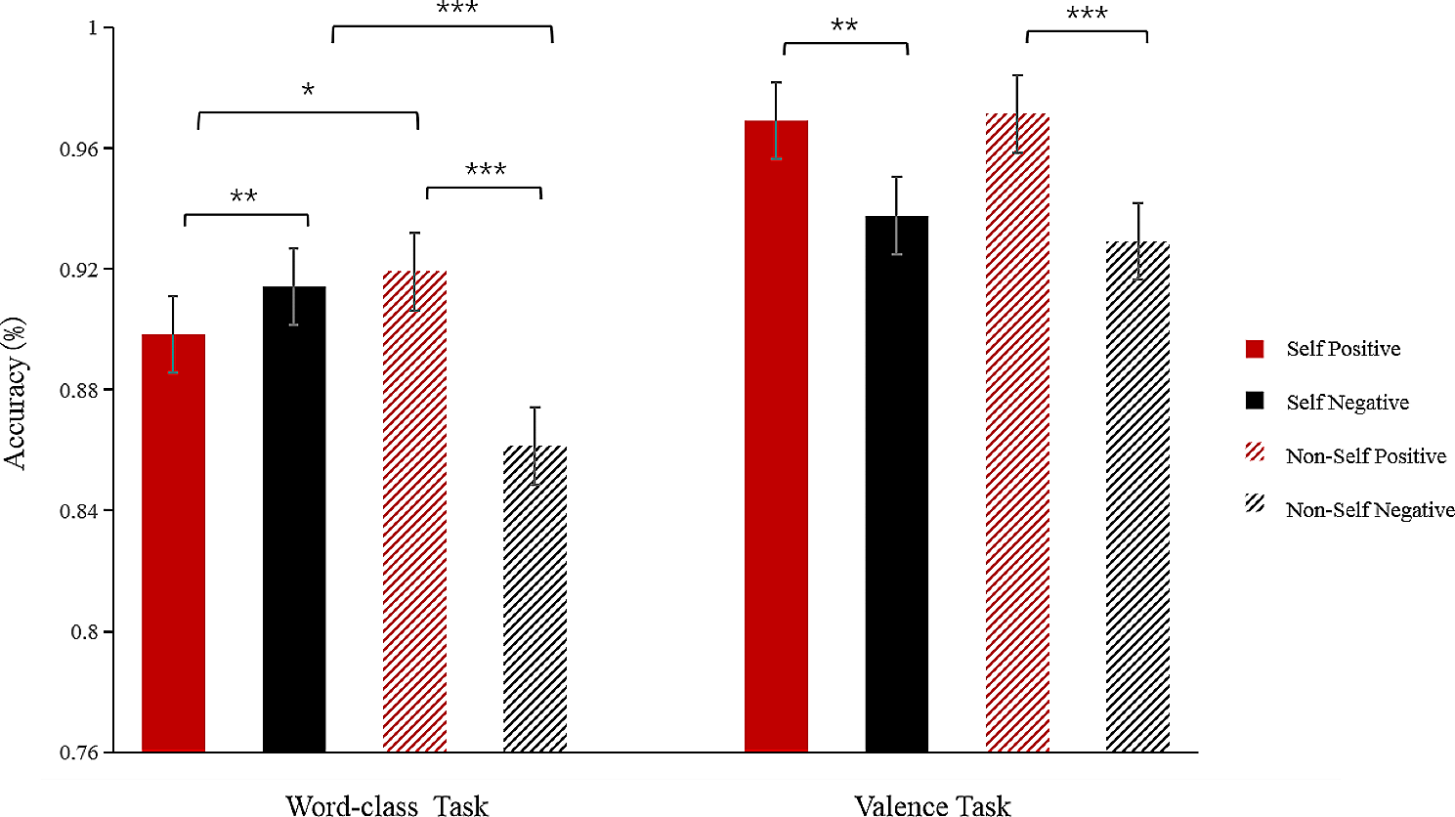
Interaction between Task type × Self-relevant cue × Emotion valence in accuracy (*<0.05;**<0.01;***<0.001)

The interaction of task type × self-relevant cue × emotion valence was significant (*F*(1, 23) = 30.83, *p* < 0.001, *η*_*p*_^*2*^ = 0.57). Further simple effect analyses revealed that in the valence task, the accuracy of positive words in the self-name condition was significantly higher than that of negative words (*F*(1, 23) = 16.13, *p* = 0.001, *η*_*p*_^*2*^ = 0.41). The accuracy of positive words was also significantly higher than negative words in the non-self name condition (*F*(1, 23) = 19.39, *p* < 0.001, *η*_*p*_^*2*^ = 0.56). In the word-class task, the accuracy of judging positive words, compared with negative words, in the self-name condition was significantly lower (*F* (1, 23) = 8.08, *p* = 0.009, *η*_*p*_^*2*^ = 0.26). In contrast, the accuracy of judging positive words, compared with negative words, in the non-self name condition was significantly higher (*F* (1, 23) = 45.22, *p* < 0.001, *η*_*p*_^*2*^ = 0.66). It was also found that the accuracy of self-relevant positive words was lower than non-self-relevant positive words (*F* (1, 23) = 6.79, *p* = 0.026, *η*_*p*_^*2*^ = 0.22). In comparison, the accuracy of self-relevant negative words was higher than non-self-relevant negative words (*F* (1, 23) = 14.36, *p* < 0.001, *η*_*p*_^*2*^ = 0.42; see Fig. 3).

### EEG results

(1) N1 (80–130 ms) and P2 (150–250 ms)

Repeated measures ANOVA revealed that the main effects and interactions of the self-relevant cue, emotion valence, and task type were insignificant for N1 and P2 (all *p*s > 0.1).

(2) EPN (150–220 ms)

**Fig. 4:**
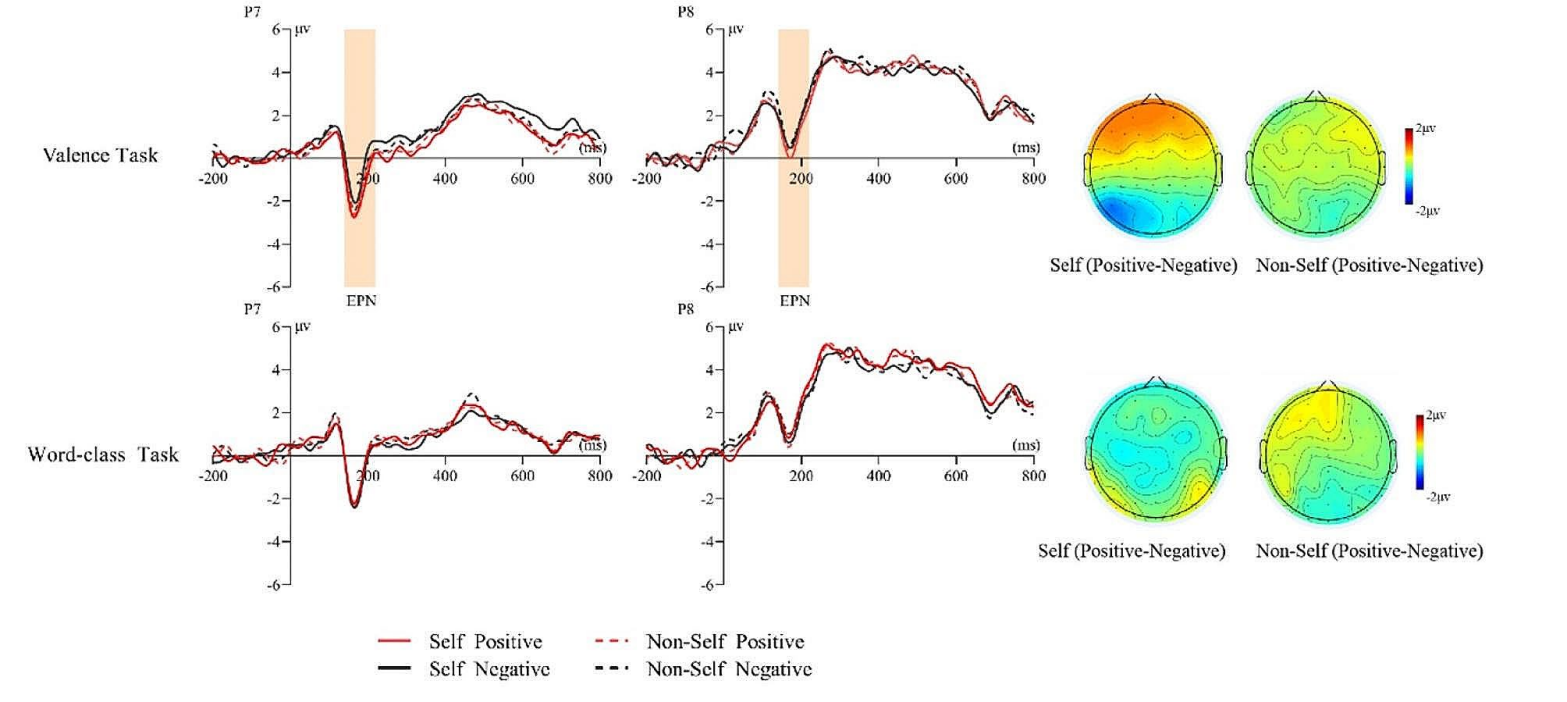
Interaction between task type × Self-relevant cue × emotion valence on EPN (150–200 ms) in the suprathreshold condition and the topographies of positive-negative difference waves with two names matching in the time window

For EPN, the main effect of threshold level was significant (*F*(1, 23) = 5.20, *p* = 0.032, *η*_*p*_^2^ = 0.18), with the suprathreshold condition (0.18 ± 0.54 µv) eliciting significantly smaller EPN amplitude than the subthreshold condition (-0.19 ± 0.50 µv). The interaction of threshold level × task type × self-relevant cue × emotion valence was significant (*F* (1, 23) = 8.08, *p* = 0.009, *η*_*p*_^*2*^ = 0.26). Simple effect analyses revealed that in the valence judgment task, self-relevant positive words (-0.38 ± 0.53µv) elicited larger EPN amplitude than self-relevant negative words (0.24 ± 0.62µv) in the suprathreshold condition (*F*(1, 23) = 9.32, *p* = 0.006, *η*_*p*_^2^ = 0.29)(see Fig. 4).

(3) N400 (300–400 ms)

For N400, the main effect of threshold level was significant (*F*(1, 23) = 12.27, *p* = 0.002, *η*_*p*_^2^ = 0.35). The suprathreshold condition (-6.53 ± 0.80 µv) elicited larger N400 amplitudes than the subthreshold condition (-5.08 ± 0.75 µv). The interaction of threshold level × self-relevant cue × emotion valence was significant (*F*(1, 23) = 6.73, *p* = 0.016, *η*_*p*_^*2*^ = 0.23). Simple effect analyses revealed that in the suprathreshold condition, self-relevant positive words (-7.09 ± 0.82 µv) elicited larger N400 amplitude than non-self-relevant positive words (-6.41 ± 0.80 µv) (*F* (1, 23) = 6.77, *p* = 0.016, *η*_*p*_^2^ = 0.23).

(4) LPP (450–650 ms)

**Fig. 5:**
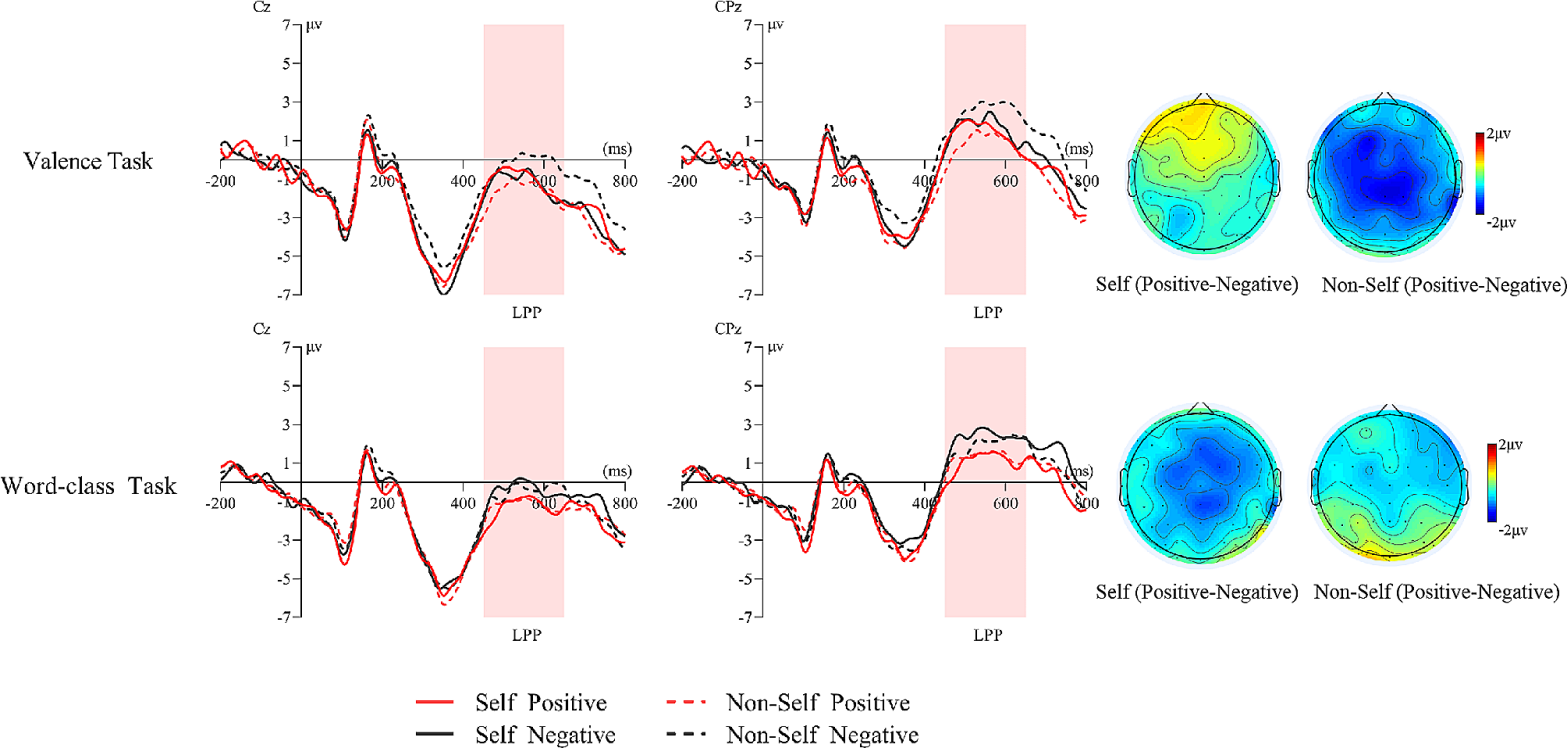
Interaction of Task type × Self-relevant cue × Emotion valence on LPP amplitude (450-650ms) in the subthreshold condition and the topographies of positive-negative difference waves with two names matching in the time window

For LPP, the main effect of emotion valence was significant (*F*(1, 23) = 20.80, *p* = 0.001, *η*_*p*_^2^ = 0.48). Positive words (0.32 ± 0.67 µv) elicited smaller LPP amplitudes than negative words (0.93 ± 0.64 µv). The interaction of threshold level × task type × self-relevant cue × emotion valence was significant (*F*(1, 23) = 4.55, *p* = 0.041, *η*_*p*_^*2*^ = 0.17). Simple effect analyses revealed that in the subthreshold condition of the valence task, non-self positive words (0.27 ± 0.66 µv) elicited smaller LPP amplitudes than non-self negative words (1.61 ± 0.60 µv); (*F*(1, 23) = 15.43, *p* = 0.001, *η*_*p*_^*2*^ = 0.40), and there was no significant difference between positive and negative words in the self-name condition (*p* = 0.696); in the subthreshold condition of the word-class task, self-relevant positive words (0.35 ± 0.74 µv) elicited smaller LPP amplitudes than self-relevant negative words (1.27 ± 0.71 µv); (*F*(1, 23) = 4.65, *p* = 0.042, *η*_*p*_^*2*^ = 0.17) (see Fig. 5).

## Discussion

This study uses the ERP technique to examine how self-relevant cues affect emotional word processing in word class and valence judgment tasks. Regarding behavioral results, in the valence judgment task, participants exhibited shorter response times and higher accuracy for self-relevant positive words than non-self-relevant positive words, demonstrating a self-positive bias. In the word-class judgment task, the accuracy of judging self-relevant positive words was significantly lower than the negative words, indicating a self-negative preference in the implicit processing of emotional information. EEG results found that in suprathreshold condition, self-relevant positive words elicited larger EPN amplitudes than negative words in the valence task, with no significant differences in the word-class task; in subthreshold condition, non-self-relevant positive words elicited smaller LPP amplitudes than negative words in the valence task, and self-relevant negative words elicited larger LPP amplitudes than positive words in the word-class task, with no significant differences in the non-self-relevant condition. These results suggest that both suprathreshold and subthreshold self-relevant information can modulate the processing of emotional information; the emotional bias differs among different tasks.

### Self-relevant positive words elicited larger EPN

The present study found that self-relevant positive words elicited larger EPN amplitudes in the valence task of the suprathreshold condition, showing a self-positive bias. In contrast, no significant differences were observed in the word-class task. EPN mainly reflects the ability of emotional words to receive more selective attention after early perceptual encoding [27]. The emotional stimuli elicit a larger EPN amplitude than neutral stimuli [26, 62]. The previous studies found that the interaction between self-relevant cues and emotional information occurred in the relatively late component of the EEG [ 37, 38,43, 45, 63]; in contrast, the present study found the effect of self-relevant cues on emotional word processing at an early stage. This may be due to the use of the familiar and stimulating information of the self-name in this experiment, leading to an earlier interaction. Unlike the use of written words such as possessive pronouns (“my”) or subjects (“I”) in previous studies, self-names have a higher social significance for individuals. They are very highly motivational and emotional [63]. Thus, even if participants are not asked to self-evaluate, they screen the emotional words first and perform more fine-grained processing of self-relevant emotional information when presented with their names. It is possible that the name rapidly activates the self-schema and its associated positive features, resulting in increased motivation to process subsequent positive stimuli. Thus, self-relevance facilitates word form analysis and lexical access to positive words, allowing them to automatically capture more attention and be more fully processed at the early encoding stage of meaning. The valence task requires individuals to fully process emotional attributes top-down to obtain more accurate and valuable dynamic information, which is rapidly linked to the pre-activated self-concept, automatically devoting more attention to positive words.

### Interaction between self and emotional information

Consistent with the results of previous studies, the present study also found an interaction between self-relevant and emotional information at the late processing stage, as reflected in the LPP component. The present study found that in the word-class task of the subthreshold condition, self-relevant negative words elicited larger LPP amplitudes than positive words, showing a self-negative bias. In contrast, there was no significant difference in the non-self state. This may be because participants in the subthreshold condition had more automatic and instinctive responses to the target stimuli. However, the experiment only required participants to make word-class judgments while ignoring emotional information. Negative words (vs. positive words) appear more difficult for individuals to inhibit, thus attracting more attention and triggering their self-protection, demonstrating a self-negative bias. The present study further confirms that LPP can sensitively reflect individuals’ implicit assessment processes of emotional stimuli.

The present study obtained opposite results in the suprathreshold condition of the valence judgment task and the subthreshold condition of the word class judgment task, demonstrating that the type of task is one of the factors affecting the interaction between self-relevant cues and emotional information. It is worth paying attention to the self-negative bias found in the subthreshold condition of word class judgment, which, on the one hand, indicates that individuals can successfully distinguish the self from others using only fewer attentional resources, activate the self-concept at the implicit level and more instinctive responses to the target stimuli; on the other hand, it also indicates that individuals have a processing advantage of self-negative bias at the implicit level. This negative bias may be because, during human evolution, the pressure for survival of the fittest has led individuals to respond more quickly, firmly, and promptly to negative stimuli, which carry a more excellent survival value than positive stimuli. Thus, negative stimuli automatically capture more attention than positive stimuli, resulting in deeper and more elaborate processing, even when there is no external task to induce individuals to devote their attention. Furthermore, we found the self-positive bias in the suprathreshold condition and self-negative bias in the subthreshold condition, suggesting that the threshold level also affects self-bias.

### Limitations and directions of further work

Although some achievements have been made in this study, it still has shortcomings and needs improvement. First, even though this study found that both suprathreshold and subthreshold self-related cues can affect the processing of emotional words, the research question and experimental design limited that subthreshold condition can only be performed before suprathreshold condition, so subthreshold and suprathreshold conditions cannot be balanced. This is an unavoidable problem. If the suprathreshold task is performed first, it must affect the later subthreshold task. In future studies, we may resolve this question by setting interference tasks or lengthening the time interval between the two tasks to balance the two experimental conditions.

Secondly, based on the fact that previous studies have used only positive and negative emotional words in examining self-relevance and emotional interactions [19, 37, 59, 64], the present study also used only these two types of words as emotional materials. Meanwhile, previous studies have shown that normal individuals’ self-default schema is usually positive; once the context establishes self-associations, the neutral words do not remain neutral, and the participants may consume more cognitive resources to process them [38]. The current study included a valence judgment task, and the presence of neutral words may have made it difficult for participants to momentarily judge their emotional valence, especially in the self-relevant condition, which triggered more in-depth processing than the negative and positive words. Therefore, the current experiment used only positive and negative emotional two-character words from the Chinese emotion word system [57]. Nevertheless, we may consider adding neutral words in future studies to more rigorously examine the issue of self-positive/negative bias.

Thirdly, ERP research has always focused on healthy subjects for emotional information processing in our brains. However, with the high incidence of emotion disorders, exploring the neural mechanism of patients with various affective disorders is necessary and significant. Researchers have used ERPs to examine the impairment of emotion perception and emotion regulation in patients with emotion disorders [16–18, 65, 66]. These research studies make a valuable attempt to solve the problem of depression and also point out the direction for the follow-up of this study. In the future, we may explore the complex interactive process of self and emotional processing in patients with emotional disorders. This serves as a beneficial attempt for disease treatment and provides supplementary practical and theoretical content for the neural mechanisms of emotional processing.

## Data Availability

The datasets used and/or analyzed during the current study are available from the corresponding author on reasonable request.

## References

[1] Pelham BW. On the nature of implicit self-esteem: the case of the name letter effect[C]//Ontario Symposium on Personality and Social Psychology. Motivated Social Perception: The Ontario Symposium. 2003; 9: 93–116.

[2] Northoff G, Heinzel A, DE GRECK M (2006). Self-referential processing in our brain–a meta-analysis of imaging studies on the self [J]. NeuroImage.

[3] Kelley W, Macrae C, Wyland C (2002). Finding the self? An event-related fMRI study [J]. J Cogn Neurosci.

[4] Qin P, Liu Y, Shi J (2012). Dissociation between anterior and posterior cortical regions during self-specificity and familiarity: a combined fMRI-meta-analytic study [J]. Hum Brain Mapp.

[5] Wuyun G, Shu M, Cao Z (2014). Neural representations of the self and the mother for Chinese individuals [J]. PLoS ONE.

[6] Fischer C, Dailler F, Morlet D (2008). Novelty P3 elicited by the subject’s own name in comatose patients [J]. Clin Neurophysiol.

[7] Hu X, Wu H, Fu G (2011). Temporal course of executive control when lying about self- and other-referential information: an ERP study [J]. Brain Res.

[8] Fan W, Chen J, Wang X (2013). Electrophysiological correlation of the degree of self-reference effect [J]. PLoS ONE.

[9] Dehaene S, Naccache L, Clec’h GL (1998). Imaging unconscious semantic priming [J]. Nature.

[10] Merikle PM, Smilek D, Eastwood JD. Perception without awareness: perspectives from cognitive psychology - ScienceDirect [J]. Cognition. 2001; 79(1–2): 115– 34. 10.1016/s0010-0277(00)00126-8 11164025

[11] Yaoi K, Osaka M, Osaka N (2015). Neural correlates of the self-reference effect: evidence from evaluation and recognition processes[J]. Front Hum Neurosci.

[12] Wang JQ, Otgaar H, Howe ML, et al. A self-reference false memory effect in the DRM paradigm: evidence from Eastern and Western samples[J]. Mem Cognit. 2019;4776–86. 10.3758/s13421-018-0851-3. 10.3758/s13421-018-0851-3 PMC6351515 30141171

[13] Balconi M, Lucchiari C (2007). Consciousness and emotional facial expression recognition subliminal/supraliminal stimulation effect on N200 and P300 ERPs [J]. J Psychophysiol.

[14] Kiss M, Eimer M (2008). ERPs reveal subliminal processing of fearful face[J]s. Psychophysiology.

[15] Schindler S, Bruchmann M, Bublatzky F. etc al. Modulation of face- and emotion-selective ERPs by the three most common types of face image manipulations[J]. Social Cognitive and Affective Neuroscience. 2019; 14(5): 493–503. 10.1093/scan/nsz027 PMC6545565 30972417

[16] Zhang DD, He ZH, Chen YM (2016). Deficits of unconscious emotional processing in patients with major depression: an ERP study[J]. J Affect Disord.

[17] Nataliie AIH, Rieke O, Krisztina N (2016). Time course of facial emotion processing in women with borderline personality disorder: an ERP study[J]. J Psychiatry Neurosci.

[18] Zhang S, Dong XF, Cui LX (2021). ERP evidence for emotional sensitivity in social anxiety[J]. J Affect Disord.

[19] Shestyuk AY, Deldin PJ (2010). Automatic and strategic representation of the self in major depression: trait and state abnormalities [J]. Am J Psychiatry.

[20] Herbert C, Junghofer M, Kissler J (2010). Event related potentials to emotional adjectives during reading [J]. Psychophysiology.

[21] Estes Z, Verges M (2008). Freeze or flee? Negative stimuli elicit selective responding [J]. Cognition.

[22] Kuchinke L, Krause B, Fritsch N, et al. A familiar font drives early emotional effects in word recognition [J]. Brain Lang. 2014;137:142–7. 10.1016/j.bandl.2014.08.007 25226214

[23] Scott G G, O’donnell P. Early emotion word processing: evidence from event-related potentials [J]. Biol Psychol. 2009;80(1):95–104. 10.1016/j.biopsycho.2008.03.010 18440691

[24] Wang L, Bastiaansen M, Yang Y, Cognitive (2013). Affect Behav Neurosci.

[25] Herbert C, Kissler J, Junghöfer M et al. Processing of emotional adjectives: evidence from startle EMG and ERPs [J]. Psychophysiology, 2006, 43(2). 10.1111/j.1469-8986.2006.00385.x 16712590

[26] Kanske P, Kotz S (2007). Concreteness in emotional words: ERP evidence from a hemifield study [J]. Brain Res.

[27] Kissler J, Herbert C, Peyk P (2007). Buzzwords: early cortical responses to emotional words during reading [J]. Psychol Sci.

[28] Lau E, Holcomb P, Kuperberg G (2013). Dissociating N400 effects of prediction from association in single-word contexts [J]. J Cogn Neurosci.

[29] Trauer S, Kotz S, Müller M (2015). Emotional words facilitate lexical but not early visual processing [J]. BMC Neurosci.

[30] De Pascalis V, Arwari B, D’antuone L (2009). Impulsivity and semantic/emotional processing: an examination of the N400 wave [J]. Clin Neurophysiology: Official J Int Federation Clin Neurophysiol.

[31] Herbert C, Junghofer M, Kissler J (2008). Event related potentials to emotional adjectives during reading [J]. Psychophysiology.

[32] Schindler S, Kissler J (2016). Selective visual attention to emotional words: early parallel frontal and visual activations followed by interactive effects in visual cortex [J]. Hum Brain Mapp.

[33] Espuny J, Jiménez-ortega L, Casado P (2018). Event-related brain potential correlates of words’ emotional valence irrespective of arousal and type of task [J]. Neurosci Lett.

[34] Zhao W, Chen L, Zhou C (2018). Neural correlates of emotion processing in word detection task [J]. Front Psychol.

[35] Han S, Ma Y (2014). Cultural differences in human brain activity: a quantitative meta-analysis [J]. NeuroImage.

[36] Hu C, Di X, Eickhoff S (2016). Distinct and common aspects of physical and psychological self-representation in the brain: a meta-analysis of self-bias in facial and self-referential judgements [J]. Neurosci Biobehav Rev.

[37] Watson LA, Dritschel B, Obonsawin MC (2007). Seeing yourself in a positive light: brain correlates of the self-positivity bias [J]. Brain Res.

[38] Fields EC, Kuperberg GR. Dynamic effects of self-relevance and task on the neural processing of emotional words in context [J]. Front Psychol. 2015;6. 10.3389/fpsyg.2015.02003. 10.3389/fpsyg.2015.02003 PMC4710753 26793138

[39] Heine SH, Lehman DR, Markus HR (1999). Is there a universal need for positive self-regard? [J]. Psychol Rev.

[40] Constable MD, Becker ML, Oh Y-I, Knoblich G (2021). Affective compatibility with the self modulates the self-prioritisation effect. Cogn Emot.

[41] Hu C-P, Lan Y, Macrae CN, Sui J (2020). Good me bad me: prioritization of the good-self during perceptual decision-making. Collabra Psychol.

[42] Vicovaro M, Dalmaso M, Bertamini M. (2022). Towards the boundaries of selfprioritization: Associating the self with asymmetric shapes disrupts the self-prioritization effect. Journal of Experimental Psychology: Human Perception and Performance. 10.1037/xhp0001036 35816564

[43] Fields EC, Kuperberg GR (2012). It’s all about you: an ERP study of emotion and self-relevance in discourse [J]. NeuroImage.

[44] Zhou H, Guo J, Ma X (2017). Self-reference emerges earlier than emotion during an implicit self-referential emotion processing task: event-related potential evidence [J]. Front Hum Neurosci.

[45] Herbert C, Herbert BM, Ethofer T (2010). His or mine? The time course of self-other discrimination in emotion processing [J]. Soc Neurosci.

[46] Zhang Q, Deng N, Jiang X (2020). The time course of self-relevance affecting emotional word processing [J]. Acta Physiol Sinica.

[47] Sander D, Grandjean D, Scherer KR (2005). A systems approach to appraisal mechanisms in emotion [J]. Neural Netw.

[48] Scherer KR, Schorr A, Johnstone T. Appraisal processes in emotion: theory, methods, research. Oxford University Press; 2001.

[49] Howe ML, Wimmer MC, Gagnon N (2009). An associative activation theory of children’s and adults’ memory illusions. J Mem Lang.

[50] Roediger HL, Balota D, Watson J. (2001). Spreading activation and arousal of false memories. In H. Roediger, J. Nairne, & A. Surprenant, editors, The nature of Remembering: Essays in Honor of Robert G. Crowder. Science Conference Series (pp 95–115). Washington, DC: American Psychological Association.

[51] Conner T, Barrett LF (2005). Implicit self-attitudes predict spontaneous affect in daily life [J]. Emotion.

[52] Koole SL, Coenen LHM. Implicit self and affect regulation: effects of action orientation and subliminal self priming in an affective priming task [J]. Self & Identity. 2014; 6(2–3): 118– 36.

[53] Lindquist KA, Gendron M, Barrett LF (2014). Emotion perception, but not affect perception, is impaired with semantic memory loss [J]. Emotion.

[54] Widen SC, Russell JA (2010). Differentiation in preschooler’s categories of emotion [J]. Emotion.

[55] Xia R, Shao H, Cui L (2021). Evidence for self-positivity bias in a subliminal self-cue: an event-related potential study [J]. Neurosci Lett.

[56] Faul F, Erdfelder E, Lang AG, Buchner A (2007). G*Power 3: a flexible statistical power analysis program for the social, behavioral, and biomedical sciences. Behav Res Methods.

[57] Wang YN, Zhou LM, Luo YJ. The pilot establishment and evaluation of Chinese affective words system [J]. Chin Mental Health J. 2008.

[58] Li S, Xu K, Xu Q, Xia R, Ren D, Zhou A (2016). Positive bias in self-appraisals from friend’s perspective: an event-related potential study. NeuroReport.

[59] Henderson LM, Baseler HA, Clarke PJ, Watson S, Snowling MJ (2011). The N400 effect in children: relationships with comprehension, vocabulary and decoding. Brain Lang.

[60] Sun Y, Sommer W, Li W (2022). How accentuation influences the processing of emotional words in spoken language: an ERP study. Neuropsychologia.

[61] Greenhouse SW, Geisser S (1959). On methods in the analysis of profile data. Psychometrika.

[62] Schacht A, Sommer W (2009). Emotions in word and face processing: early and late cortical responses [J]. Brain Cogn.

[63] Tacikowski P, Nowick AA (2010). Allocation of attention to self-name and self-face: an ERP study [J]. Biol Psychol.

[64] Yun C, Yiping (2014). Evidence for implicit self-positivity bias: an event-related brain potential study [J]. Exp Brain Res.

[65] Li YZ, Kang C, Wei ZG, et al. Beta oscillations in major depression– signalling a new cortical circuit for central executive function[J]. Sci Rep. 2017;18021. 10.1038/s41598-017-18306-w. 10.1038/s41598-017-18306-w PMC5740142 29269891

[66] Li YZ, Kang C, Qu XD et al. Depression-related brain connectivity analyzed by EEG event-related phase synchrony measure [J]. Front Hum Neurosci. 2016;(10). 10.3389/fnhum.2016.00477 PMC5035751 27725797

